# Dissecting Genotype by Environment Interactions in Moroccan Wheat: An Advanced Biplot and Heatmap Analysis Unveiling Agronomic, Quality Traits, and Genotypic Stability for Tailored Breeding Strategies

**DOI:** 10.3390/plants13081068

**Published:** 2024-04-10

**Authors:** Oussama Hnizil, Aziz Baidani, Ilham Khlila, Mouna Taghouti, Nasserelhaq Nsarellah, Ali Amamou

**Affiliations:** 1 Laboratory of Agrifood and Health, Faculty of Sciences and Techniques, Hassan First University of Settat, P.B. 577, Settat 26000, Morocco; aziz.baidani@uhp.ac.ma (A.B.); ilhamo.khlila@gmail.com (I.K.); 2 Research Unit of Plant Breeding and Genetic Resources Conservation, Regional Center of Agricultural Research of Settat, National Institute of Agricultural Research, P.B. 589, Settat 26000, Morocco; nsarellah@yahoo.com; 3 Research Unit of Plant Genetic Resources and Plant Breeding, National Institute for Agronomic Research, P.B. 6356, Institutes 1010, Rabat 10101, Morocco; taghoutimouna@yahoo.fr

**Keywords:** genotype × environment (G × E), durum wheat, agronomic traits, quality traits, breeding strategies, agro-climatic zones

## Abstract

This five-year study (2016–2021) across diverse Moroccan agro-climatic zones investigated genotype by environment (G × E) interactions in wheat, focusing on variations in agronomic traits and quality attributes such as protein and gluten content. Significant environmental effects were observed on key traits, like yield, thousand kernel weight (TKW), and spikes per square meter (Spk/m^2^), highlighting environmental factors’ role in wheat yield variability. In the Tassaout (TST) location, notable genotypic effects emerged for traits like biomass, underscoring genetic factors’ importance in specific contexts, while in Sidi El Aidi (SEA) and Marchouch (MCH), genotypic effects on yield and its components were predominantly absent, indicating a more substantial environmental influence. These findings illustrate the complexity of G × E interactions and the need for breeding strategies considering genetic potential and environmental adaptability, especially given the trade-offs between yield enhancement and quality maintenance. Insights from the biplot and heatmap analyses enhanced the understanding of genotypes’ dynamic interactions with environmental factors, establishing a basis for strategic genotype selection and management to optimize wheat yield and quality. This research contributes to sustainable wheat breeding in Morocco, aligning with global efforts to adapt wheat breeding strategies to changing climatic conditions.

## 1. Introduction

Durum wheat (*Triticum durum*) is a crucial cereal crop, particularly in the Mediterranean regions, including Morocco, where it plays a vital role in food security and the rural economy. The performance of durum wheat varieties is highly influenced by the complex interactions between genotypes (G) and environmental (E) factors, collectively termed genotype by environment (G × E) interactions. These interactions have been a focal point in agronomic research, as they significantly impact the key traits of economic importance, such as yield, grain quality, and resilience to changing climatic conditions [1,2].

The complexity of G × E interactions necessitates a meticulous examination to garner substantial insights for tailoring breeding strategies. Previous research efforts have underscored the complexity of G × E interactions, which requires a thorough examination to garner insightful observations for tailoring breeding strategies. Previous research efforts have highlighted the significant impact of climatic variables on wheat performance, revealing the marked influence of environmental conditions on vital agronomic traits, such as yield, thousand kernel weight (TKW), and spikes per square meter (Spk/m^2^) [3]. These findings underscore the imperative for a genotype-specific approach in breeding programs to maximize the inherent potential of different genotypes under varied environmental conditions.

Furthermore, advancements in statistical methodologies have facilitated a deeper understanding of these interactions. Techniques like biplot analyses and heatmap visualizations have been pivotal in unraveling complex genotype–environment relationships, assisting in evaluating and selecting genotypes with optimal performance and stability across diverse environmental conditions [4,5,6]. Exploring environmental discrimination and representativeness through biplot analysis has further enriched our comprehension of how various environments affect the primary traits of interest, specifically yield and protein content in wheat [7].

Identifying and understanding stable genotypes under fluctuating environmental conditions have been active research areas. In an era of changing environmental dynamics, pinpointing genotypes with consistent performance across diverse agro-climatic zones is paramount for developing resilient and high-yielding varieties [8,9].

The goal of the present study, titled “Dissecting Genotype by Environment Interactions in Moroccan Wheat: An Advanced Biplot and Heatmap Analysis Unveiling Agronomic, Quality Traits, and Genotypic Stability for Tailored Breeding Strategies”, was to conduct an exhaustive analysis of G × E interactions over five wheat-growing seasons in different agro-climatic zones in Morocco. Using advanced statistical methodologies, this study sought to illuminate the varying performance of wheat genotypes across changing environmental settings, assess the influence of environmental conditions on primary agronomic and quality traits, and determine genotypic stability across various environments. This initiative offers actionable insights for breeding initiatives focused on cultivating high-yield, resilient wheat varieties compatible with various Moroccan agro-climatic zones. Such efforts are critical in enhancing wheat productivity and fortifying its resilience against unpredictable and shifting climatic scenarios.

In line with these objectives, this study presents an in-depth analysis of genotype–environment interactions and their implications for agronomic and quality traits. It establishes a sturdy analytical foundation poised to inform future breeding programs aiming to elevate yield and quality traits amidst fluctuating environmental settings. By meticulously examining genotype-specific responses to environmental factors, this study sets a solid precedent for nuanced interpretations and applications in agronomic practices, marking a significant contribution to the wheat breeding conversation in Morocco.

## 2. Materials and Methods

### 2.1. Research Locations and Climatic Variables

This study spanned five growing seasons (S): 2016–2017, 2017–2018, 2018–2019, 2019–2020, and 2020–2021, and was carried out at three distinct research installations of the National Institute of Agricultural Research (INRA) of Morocco. Detailed climate characteristics of each installation are provided in Table 1.

### 2.2. Experimental Framework

In this field study, we implemented a split-plot design across 90 individual plots to meticulously examine the responses of different crop genotypes under varying nitrogen levels. This design was integral in replicating the complex conditions encountered under agricultural settings. Each plot was distinctively managed to represent various combinations of genotypes and nitrogen treatments.

For a more targeted analysis, we focused on a subset of 30 plots, all receiving the same nitrogen dose. This selection was made to facilitate an in-depth investigation of genotype and environmental interactions under uniform nitrogen conditions. Maintaining a consistent nitrogen application across these plots was essential to minimize variability due to differences in nitrogen levels, thereby enabling a precise assessment of how genotypes interact with environmental factors.

The data from these 30 plots were analyzed using a two-way analysis of variance. This statistical method is well-suited for our study’s objective, as it allows for an in-depth evaluation of the interaction effects between crop genotypes and environmental conditions under a consistent nitrogen regime, here defined as the growing season in a particular location. The use of the two-way ANOVA aids in the detailed and accurate interpretation of these interactions, which is crucial for understanding the adaptability and performance of different genotypes under a controlled nutrient environment.

The model employed for this analysis is structured as follows:

$$Y_{ijk}=\mu +\alpha_{i}+\beta_{j}+{\left(\alpha \beta \right)}_{ij}+\varepsilon_{ijk}$$

where $Y_{ijk}$ represents the observed response for the i-th genotype, j-th environment (specifically, the growing season in a location), and k-th replication; μ is the overall mean across all genotypes, environments (growing seasons in locations), and replications; α_i_ and β_j_ denote the main effects of the genotype and environment (growing season in a location), respectively; (αβ)_ij_ captures the interaction effect between the genotype and the environment (growing season in a location); and $\varepsilon_{ijk}$ represents the random error component.

A detailed description of the genotypes that were used is presented in Table 2.

#### Characteristics of Experimental Plots and Agronomic Protocols

Each experimental plot covered an area of 2.7 square meters, measuring 2.5 m in length and 1.08 m in width. Standard agronomic practices, encompassing soil treatment, weed control, and irrigation, especially at the Tassaout (TST) location, were implemented to ensure optimal growth conditions. Sowing occurred in mid-November using a Wintersteiger plot seeder, with planting schedules tailored to align with the unique climatic conditions of each research location.

### 2.3. Data Collection and Trait Measurement

Data were collected on various agronomic and quality traits, adhering to stringent protocols and international standards. Crop yield was measured in grams per 2.7 square meters and converted into quintals per hectare for consistency. Above-ground biomass was evaluated pre-harvest and quantified in kilograms. The thousand kernel weight (TKW) was determined using an electronic grain counter compliant with NF V03-702 and ISO 520 standards. Spikes per square meter (Spk/m^2^) were counted from designated 1-square-meter sample areas within each plot. Additionally, quality traits, such as protein content, gluten levels, and baking strength, were meticulously assessed using Chopin Technologies’ Infraneo near-infrared spectroscopy (NIRS). The device was calibrated regularly to ensure accuracy at the INRA facility in Rabat. To affirm the reliability of the NIRs’ measurements, cross-validations were conducted using a calibrated FOSS Infratec NIR analyzer at the INRA facility in Settat. The outcomes derived from the NIR instruments were stringently compared and authenticated against the Kjeldahl method, guaranteeing the credibility and consistency of the reported findings.

### 2.4. Statistical Analysis

In the process of data processing and preliminary analysis, Microsoft Excel was employed for basic data cleaning and preliminary exploratory analyses.

The inferential statistics in our study were conducted using Minitab 18, specifically through a two-way analysis of variance (ANOVA). This approach is in line with the methodologies detailed in the “Minitab Cookbook” [10], which provides an in-depth explanation of conducting complex ANOVAs using Minitab. To further substantiate our findings, post-hoc comparisons of means were conducted using the Tukey’s honest significant difference (HSD) method. This method, as detailed in the ‘Minitab Handbook’ [11], is crucial for determining which specific means differ when the null hypothesis in ANOVA is rejected. It effectively controls the family-wise error rate, ensuring an honest significance level across all pair-wise tests, and is particularly well-suited for analyses conducted in Minitab.

Further, this study utilized the R programming language for calculating stability parameters and creating statistical visualizations. The stability parameters included the Francis cumulative, Wricke’s ecovalance (W), and Shukla’s stability variance (σ2). The Francis cumulative method was based on the work of Francis and Kannenberg [12] in their study on yield stability, which provided a descriptive method for grouping genotypes. Wricke’s ecovalance (W) was derived from the methodology proposed by Wricke [13] to capture the ecological range in field experiments. Lastly, Shukla’s stability variance (σ2) was adopted from Shukla’s [14] work, which focused on the statistical aspects of partitioning the genotype–environmental components of variability. These methods were integral in analyzing the stability of various genotypes in our study.

Pearson’s correlation matrix was calculated using R to evaluate the linear relationships between various variables. This analysis was guided by the approach described in “Applying Statistical Methods to Library Data Analysis” [15], which emphasizes the application of statistical methods, including Pearson’s correlation, in data analysis using R.

Lastly, this study incorporated advanced multivariate analyses using R, including discrimination vs. representativeness plots, ranking environments’ analyses, mean vs. stability biplot analyses, and which won where/what biplot analyses. These analyses, based on principal component analysis or singular value decomposition, followed the methodologies outlined by Tonk et al. [16]. This approach facilitated a deeper and more nuanced understanding of the interactions between genotypes and environments.

## 3. Results

### 3.1. Descriptive Statistics for Yield and Yield Components across Multiple Locations

The agronomic characteristics of five genotypes, coded V1 (Faraj), V2 (Itri), V3 (Karim), V4 (Luiza), and V5 (Nassira), were evaluated across five successive growing seasons (from 2016–2017 to 2020–2021) at the experimental station Sidi El Aïdi (SEA). This study defined “environment” as the varying growing seasons at the SEA location. A comprehensive two-factor analysis of variance (ANOVA) was employed to assess the agronomic traits of these genotypes over the different seasons. Post-hoc Tukey tests were conducted, identifying significant differences at the 95% confidence level, as detailed in Table 3. This analysis provided an insightful depiction of how genotypes V1 through V5 responded to environmental variations across seasons.

The ANOVA findings highlighted the considerable impact of environmental conditions on key agronomic traits. Specifically, the analysis revealed a significant environmental effect on yield (*p* ≤ 0.001). This pattern was also reflected in other traits, such as thousand kernel weight (TKW), spikes per square meter (Spk/m^2^), and grains per square meter (G/m^2^), all showing similarly significant environmental influences. Notably, the environmental factors did not significantly impact biomass nor the number of grains per spike (G/S).

Regarding the genotypic impact, it was observed that the genotype had a significant exclusive effect on the TKW (*p* ≤ 0.05). Traits like yield, biomass, Spk/m^2^, G/S, and G/m^2^ did not exhibit significant differences attributable to genotypic variations. Furthermore, the interaction between genotype and environment (G * E) demonstrated statistical significance for the TKW (*p* ≤ 0.05) and Spk/m^2^ (*p* ≤ 0.01), indicating variability in the performance of different genotypes across the seasons for these traits.

In examining the annual yield performance, genotypes V5 (Nassira), V1 (Faraj), and V4 (Luiza) demonstrated notable variations across different growing seasons. These variations showcased a pronounced increase in yield of 80.7% from the 2016–2017 season to the 2017–2018 season, followed by a decrease of 59.6% in 2018–2019. This trend continued with a significant decline of 65% in 2019–2020 and an extraordinary rebound of 250% in the 2020–2021 season. It is essential to highlight that these yield changes were predominantly influenced by environmental factors rather than solely by genetic attributes of these genotypes.

Regarding analyzing other agronomic traits, the biomass evaluation revealed no significant differences related to genotype or environmental factors. Concurrently, thousand kernel weight (TKW) exhibited notable interactions between the genotype and environment. Among the genotypes, V5 and V4 distinguished themselves in biomass performance, and V5 and V3 in TKW across varying seasons. Despite these observations, it is essential to interpret any direct relationship between biomass and the TKW with caution, considering the complexity of the underlying interactions.

Additionally, this study observed variability in spikes per square meter (Spk/m^2^), which was attributed to both environmental factors and the genotype–environment (G * E) interaction. This variability and the observed patterns in grains per spike (G/S) and grains per square meter (G/m^2^) emphasize the genotypes’ capacity to adapt to changing environmental conditions. However, it is critical to acknowledge that the ANOVA results indicated no significant influence of the genotype, environment, or their interaction on G/S and G/m^2^. This finding indicates that the observed variations in these traits across different seasons at the SEA location cannot be solely attributed to genetic or environmental factors but are likely the result of a more intricate interplay between these elements.

Expanding on the insights gathered from the Sidi El Aidi (SEA) location, our analysis was extended to the experimental station Marchouch (MCH). The comprehensive study at MCH offers a deeper understanding of how environmental factors significantly impact agronomic traits across different growing seasons. The results from MCH, detailed in Table 4, underscore the notable influence of both genotype and environmental conditions, and the interactions between these factors on vital agronomic traits.

Yield and biomass exhibited notable annual fluctuations. For instance, yield saw a significant increase of 28.1% between the 2016–2017 and 2017–2018 seasons, followed by a dramatic decline and a resurgence in subsequent years. Biomass trends showcased similar variations.

The thousand kernel weight (TKW) and spikes per square meter (Spk/m^2^) also demonstrated significant oscillations across the study period, reflecting environmental variability.

The grains per spike (G/S) showed dynamic fluctuations with significant genotype (Gen) * environment (Env) interaction effects, as indicated by the analysis of variance. These interactions underscore the complexity of agronomic traits influenced by both genetic and environmental factors. 

What was particularly striking was the significant effect of genotype on grains per square meter (G/m^2^). A compelling example was observed during the 2020–2021 season, where the V1 and V5 genotypes showed remarkably different G/m^2^ values under the same environmental conditions. V1 recorded 14,693 G/m^2^, whereas V5 demonstrated a significantly higher 23,895 G/m^2^ value. This difference was statistically significant according to the ANOVA and was confirmed with the Tukey’s post-hoc test, highlighting the substantial impact of genetic factors on this trait.

**Figure 1:**
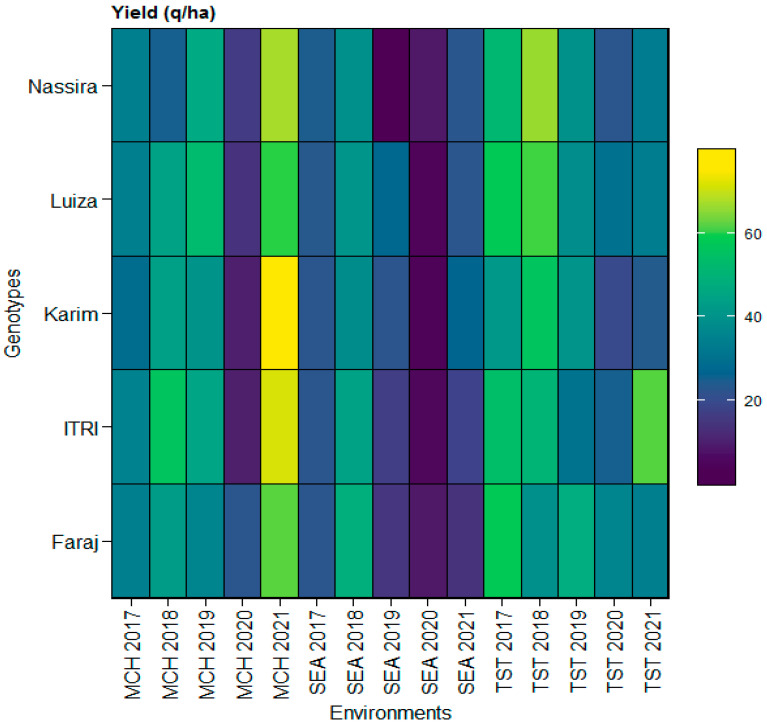
Heatmap illustrating yield variations across genotypes (Faraj, Itri, Karim, Luiza, and Nassira) and environments (SEA 2017–2021, MCH 2017–2021, and TST 2017–2021).

Incorporating the heatmap analysis, it became discernible that the yield variations were intricately linked to both genotype and environmental conditions at each location. The heatmap provided a comprehensive visual summary of the yield dynamics across different genotypes under varying environments from the SEA, MCH, and TST locations the years from 2017 to 2021. For instance, genotypes like Faraj (V1) and Itri (V2) demonstrated consistent yield performances under the SEA environments, corroborating the quantitative findings from SEA. Conversely, the heatmap also vividly captured the pronounced yield variations exhibited by genotypes like Luiza (V4) and Nassira (V5) in the TST and MCH environments, reinforcing the observed fluctuations in yield in these locations. This integrative approach combined quantitative analysis and visual representation, enabling a more nuanced understanding of the multifaceted interactions between genotype, environment, and yield. It also offered valuable insights into different genotypes’ adaptive capacities and yield potentials (V1–V5) under varied environmental conditions, as depicted in Figure 1.

The two-way analysis of variance (ANOVA) conducted across different locations, including the TST station, revealed distinct patterns in the effects of genotype and environment on agronomic traits. The results for the TST location, as detailed in Table 5, show significant genotypic effects on biomass, thousand kernel weight (TKW), spikes per square meter (Spk/m^2^), and grains per spike (G/S), all at *p* < 0.01. These findings at TST starkly contrast with other environments like SEA and MCH, where such genotypic effects were less pronounced.

At TST, the significant genotypic effects on biomass also highlighted a notable interaction between genotype and environment (genotype * environment). For instance, the V3 genotype demonstrated considerable variation in biomass values, increasing from 79.5 q/ha in 2017 to 152.5 q/ha in 2018. Similarly, the V2 genotype fluctuated from 107.5 q/ha in 2019 to 59.75 q/ha in 2020. These changes underscore the significant interaction effect, where different genotypes responded uniquely to environmental conditions over the years.

The environmental impact was notably significant on all traits except for the grains per spike (G/S) trait. For instance, the average biomass at TST showed considerable fluctuations over the years, reflecting environmental variability. In 2017, the average biomass was 105.3 q/ha, increasing to 140.83 q/ha in 2018, then decreasing to 120 q/ha in 2019, and further declining to 74.99 q/ha in 2020. However, a significant recovery was observed in 2021, with the average biomass rising to 146.18 q/ha. These variations align with the ANOVA results’ highly significant environmental differences (*p* < 0.001).

**Figure 2:**
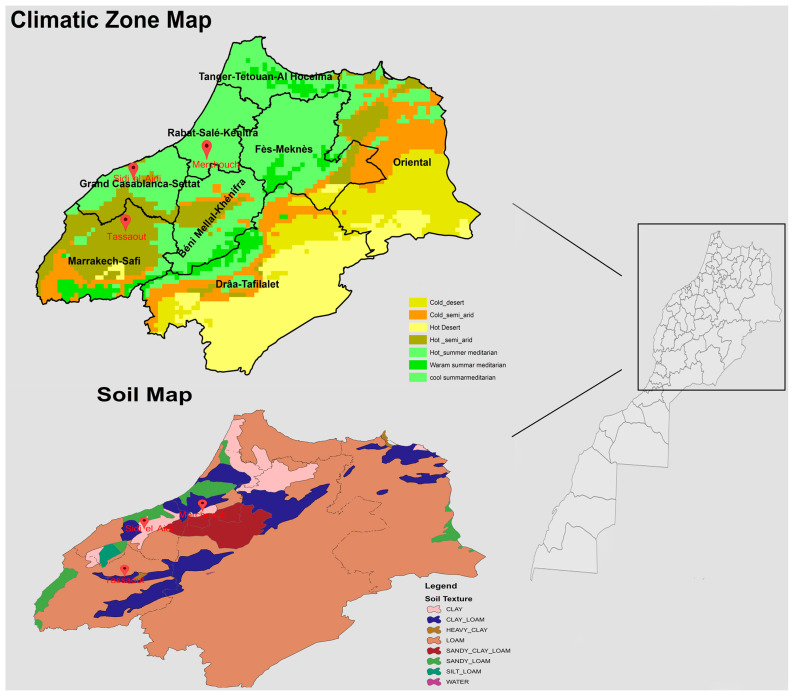
Climatic zone map and soil map of Morocco.

Further analyses, as detailed in Table 1, elucidate the role of soil type in influencing wheat growth. The soil types at SEA (Vertisol), MCH (Cambisol), and TST (Alfisol) significantly impact wheat growth. Additionally, as indicated in Figure 2, soil texture varies from clay at SEA to clay loam at MCH and TST. These variations in soil texture affect water retention, aeration, and nutrient availability, influencing wheat traits like biomass, TKW, Spk/m^2^, and G/S.

The variability in precipitation from 2016 to 2021 also showed a substantial effect on wheat yields and other traits. For example, SEA experienced a precipitation low of 210 mm in 2018–2019 and a high of 505 mm in 2017–2018. These fluctuations in precipitation directly affect wheat yields and biomass, with increased precipitation typically leading to higher yields.

The distinct response of different wheat genotypes under varying environmental conditions, including soil type, texture, and precipitation, underscores the need for targeted breeding programs. Breeding programs should focus on genetic potential and environmental adaptability to optimize wheat production under specific agroecological zones.

The complex interplay between soil, the climate, and genotype is crucial in determining the agronomic traits of wheat. The significant variations observed across locations highlight the need for context-specific agronomic practices and breeding strategies. Understanding these dynamics can aid in developing wheat varieties that are resilient and high-yielding under varying environmental conditions. These findings from TST, in particular, underscore the importance of targeted breeding and management strategies that consider both genetic potential and environmental adaptability.

### 3.2. Discrimination vs. Representativeness Biplot Analysis

**Figure 3:**
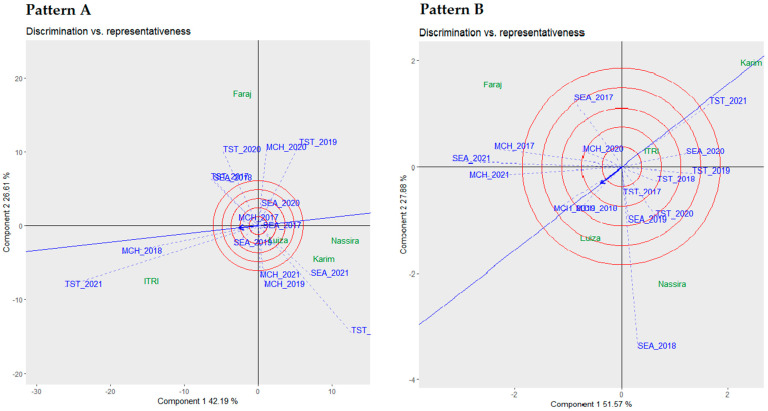
Comparative biplot analysis of environmental discrimination and representativeness: yield (**Pattern A**) and protein (**Pattern B**).

In Figure 3, discrimination vs. representativeness biplot analyses are depicted, elucidating the discriminative capacities and representativeness of 15 scrutinized environments, focusing on yield and protein as the principal traits of interest.

Pattern A of Figure 3 details the analysis based on yield data. The biplot indicates that the first component accounts for 42.19% of the variability, while the second component explains 26.61%, representing a significant proportion of the total variability. TST 2021 stood out as the most discriminating environment, in contrast to SEA 2017, which was marked as the least discriminating. On the other hand, MCH 2018 was identified as the most representative environment, capturing a broad spectrum of environmental variables. Notably, the variety of Luiza stood out, highlighting its adaptability across varied environmental conditions. Such nuanced observations are crucial for advancing understanding in environmental studies, mainly when focusing on yield variability across diverse environments.

Pattern B of Figure 3 showcases the analysis with protein as the primary trait. This biplot was proven to be pivotal in assessing the distinguishing abilities of the environments, as measured via the environmental vector’s length. Of the 15 environments analyzed, SEA 2018 emerged as the most discriminating environment, followed by SEA 2021, MCH 2021, and TST 2021, while TST 2017 was perceived as the least discriminating. This biplot also highlighted that MCH 2018 was the most representative environment, succeeded by MCH 2019 and TST 2021. The variety Itri occupied a central position in this biplot, suggesting consistent representation across the examined environments.

Given the findings from the discrimination vs. representativeness biplot analysis, a deeper exploration into the hierarchical framework of different environments becomes essential, as discussed in the following section.

### 3.3. Ranking Environments via Biplot Analysis

**Figure 4:**
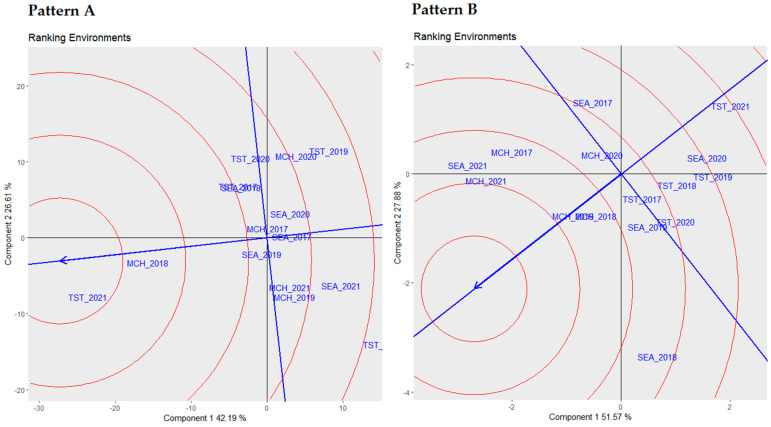
Hierarchical environmental ranking via biplot analysis: evaluating alignment with ideal conditions for yield (**Pattern A**) and protein (**Pattern B**) under diverse environments.

Figure 4 displays the ranking environment biplots, shedding light on the hierarchical structure of different environments based on yields and proteins. The ideal environment occupies a central position within concentric circles, acting as the benchmark for the target environment.

Pattern A of Figure 4 delves into the biplot analysis for yield. The concentric circles in this biplot represent varying degrees of proximity to the ideal environment. Under this setting, environment TST 2021 was deemed to be the closest to the ideal environment, indicating its superior alignment with the ideal conditions for yield. After that, MCH 2018 emerged as the second-closest environment to the ideal, demonstrating its significant alignment with the optimal conditions for yield.

Conversely, pattern B of Figure 4 details the biplot analysis centered on proteins. The concentric circles illustrate the environments’ alignment with the ideal conditions for proteins. Here, environment MCH 2021 was highlighted as the closest to the ideal environment, showing its optimal alignment with the desired conditions for proteins. Following this, MCH 2019 and MCH 2018 were designated as the second- and third-closest environments to the ideal, respectively, in that order, indicating their strong alignment with the optimal conditions for proteins.

Upon determining the hierarchical structure of the environments based on yields and proteins, our ensuing task involved evaluating the stability of the genotypes across these diverse environments. This progression introduced a comprehensive assessment of genotypic stability.

### 3.4. Comprehensive Stability Analysis

In our comprehensive analysis, as detailed in Table 6, we employed three robust statistical metrics, namely Francis cumulative values, Wricke’s ecovalence (W), and Shukla’s stability variance (σ2), to evaluate the stability of five distinct genotypes across multiple environments. The choice of multiple stability parameters was driven by the need to capture the multifaceted nature of genotype–environment interactions. Each metric offers unique insights, ranging from mean performance (Francis cumulative values) to interaction variances (Wricke’s ecovalence and Shukla’s stability variance). This provides a more nuanced and complete understanding of genotypic stability. The genotypes were ranked based on these stability metrics to provide an integrated view of their performance.

Contrary to our initial expectations, “V3” emerged as the most stable genotype according to Shukla’s stability variance (σ2) with a score of 400, leading to its first-place ranking (GR = 1) in this category. However, it displayed less stability in Francis cumulative values with a score of 34, placing it lower in that ranking.

“V1”, on the other hand, demonstrated exceptional stability under Wricke’s ecovalence (W) with a score of 3607,727, meriting the top rank in this specific metric. Interestingly, “Faraj” also showed strong performance in Shukla’s stability variance (σ2), ranked second with a score of 457, indicating its overall stability across varied conditions.

Meanwhile, “V4” exhibited noteworthy stability in Francis cumulative values, leading the category with a score of 49, suggesting a solid adaptability to different environments. In Shukla’s stability variance (σ2), “V4” maintained a solid performance with a score of 333. However, it ranked third in this metric, indicating specific stability traits that may be advantageous under certain environmental scenarios.

These findings highlight that while specific genotypes like “V3” and “V1” show strong stability across different metrics, each genotype possesses unique stability characteristics. This suggests the potential for specific genotypic advantages under certain environmental conditions, underscoring the need for a nuanced approach in selecting genotypes for diverse agricultural settings.

Upon establishing the stability of these genotypes, it was crucial to comprehend their mean performance in correlation with their stability, which was visually represented in the following biplots.

### 3.5. Mean vs. Stability Biplot Analysis

**Figure 5:**
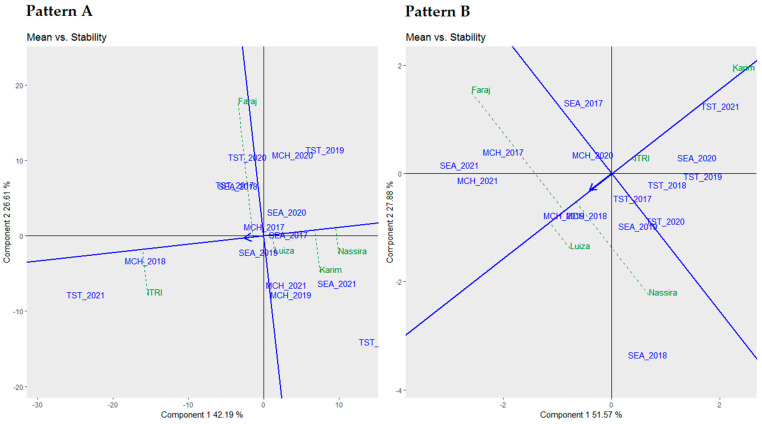
Comparative biplot analysis of genotypic mean performance and stability: yield (**Pattern A**) and protein content (**Pattern B**).

Figure 5 displays the mean vs. stability biplots to rank the examined genotypes hierarchically based on their mean performance and stability regarding yield and protein.

Pattern A of Figure 5 illustrates the biplot analysis for yield. This biplot intended to assess and rank the genotypes based on their mean yield performance and stability across various environments. From this visualization, Luiza emerged as the most stable genotype, followed in sequence by Nassira, Karim, and Faraj regarding stability. Conversely, Itri displayed a higher yield mean when contrasted with the overall mean performance, while Nassira and Karim registered lower yield means relative to the overall mean performance.

Pattern B of Figure 5 addresses the biplot analysis centered on proteins. This biplot endeavored to categorize the tested genotypes based on their mean performance and stability in protein content. Through this perspective, Itri was identified as the most stable genotype, with Karim, Luiza, Nassira, and Faraj successively aligned in terms of stability. Conversely, Luiza, Faraj, and Nassira exhibited higher protein means compared to the overall mean performance, while Itri and Karim presented lower protein means compared to the overall mean performance.

The insights that were gained from the mean vs. stability biplots prepare a foundation for further analysis of the superior performance of the genotypes under distinct environments, leading us to the “Which Won Where/What” biplot analysis.

### 3.6. Which Won Where/What Biplot Analysis

**Figure 6:**
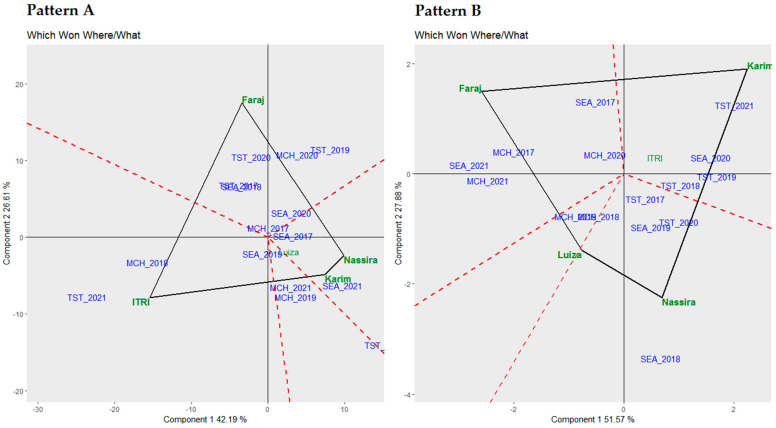
Detailed biplot analysis of genotypic superiority in varied environments: yield (**Pattern A**) and protein content (**Pattern B**).

Figure 6 displays the “Which Won Where/What” polygon patterns, highlighting the superior performance of various genotypes under distinct environments focusing on yield and protein.

Pattern A of Figure 6 depicts the analysis for yield. This polygon pattern identified the leading genotypes of each environment, shedding light on their adaptability and performance under certain conditions. Itri was the top-performing genotype under environments MCH 2018 and TST 2021, emphasizing its adaptability and high yield under these settings. On the other hand, Faraj emerged as the best-performing genotype in TST 2020, MCH 2020, TST 2019, TST 2017, and SEA 2018, signifying its strong performance and versatility across a diverse set of environments. Furthermore, Nassira and Karim were highlighted as the leading performers in SEA 2021, demonstrating their exceptional yields under this setting.

Pattern B of Figure 6 offers a contrasting view, concentrating on proteins. The polygon pattern here denoted the leading genotypes of each environment based on protein content. Faraj was the top-performing genotype in SEA 2017, MCH 2017, SEA 2021, and MCH 2020, underscoring its high protein content under these settings. Concurrently, Karim was singled out as the best-performing genotype in TST 2021, SEA 2020, TST 2018, and TST 2019, reflecting its premium protein content and adaptability under these defined contexts.

### 3.7. Descriptive Statistics for Quality Traits across Environments

At the SEA location, significant environmental effects (*p* < 0.001) were observed on protein content, along with notable genotype–environment interaction effects (*p* < 0.001). The protein content in variety V1 exhibited marked fluctuations, as shown in Table 7. Specifically, the protein content ranged from 19.46% in 2017, decreased to 13.80% in 2020, and rose to 20.90% in 2021. Comprehensively detailed in Table 7, these variations underscore the combined influence of environmental conditions and genotype–environment interactions on protein content.

A genotypic influence (*p* < 0.01) was observed regarding gluten content at SEA. It was also significantly influenced via both the environment (*p* < 0.001) and the genotype–environment interaction (*p* < 0.001), and the 2016–2017 growth season marked the peak of mean gluten content at 47.65%, where both Faraj and ITRI excelled, showcasing values of 49.63% and 49.9%, respectively. The lowest mean was documented in 2019–2020, settling at 36.0%, with Faraj recording the lowest value of 31.26%.

Baking strength at SEA revealed significant distinctions due to genotype (*p* < 0.01) and was profoundly shaped by the environment (*p* < 0.001), along with the genotype–environment interaction (*p* < 0.001). During the 2016–2017 growth span, the mean baking strength stood at 449.03 W, with Faraj ascending to the pinnacle spot with 494.29 W. A decline followed in subsequent years, culminating in the nadir in 2019–2020 at an average of 332 W. Nevertheless, the 2020–2021 season marked a revival, averaging at 418 W, with Faraj recording a notable strength of 461.83 W.

For the Marchouch (MCH) site, protein content displayed a paramount genotype influence (*p* < 0.001) and was strongly influenced by environmental factors (*p* < 0.001), as detailed in Table 8. However, the interplay between genotype and environment was found to be insignificant (ns). During the 2016–2017 growth season, the mean protein content for all varieties was 20.16%, with Faraj attaining the peak value of 22%. A marked decrease occurred in 2017–2018, causing the mean protein content to drop to 15%. The 2020–2021 season recorded a mean protein content of 16.64%, with Faraj showing a significant resurgence to 18.5%.

Regarding gluten content at MCH, a dominant genotypic impact (*p* < 0.001) was identified, coupled with a pronounced environmental influence (*p* < 0.001). The interaction between genotype and environment was present, though lower in its magnitude (*p* < 0.05). The 2016–2017 growth span marked the apex in mean gluten content at 59.76%, with both Faraj and Luiza making a solid presence, sequentially registering values of 64.82% and 63%, respectively. A trough was hit in 2017–2018, pinning the mean gluten content at 36%, with Karim’s value dwindling to 32.43%.

Baking strength in MCH demonstrated an overarching genotypic impact (*p* < 0.001) and was decisively molded through environmental factors (*p* < 0.001). The interrelation between genotype and environment stood out emphatically (*p* < 0.001). During the 2016–2017 growth phase, the mean baking strength settled at 549.22 W, with Faraj elevating to a peak strength of 622.86 W. Subsequent growth periods witnessed a waning, bottoming out in 2017–2018 with a mean value of 316 W.

**Figure 7:**
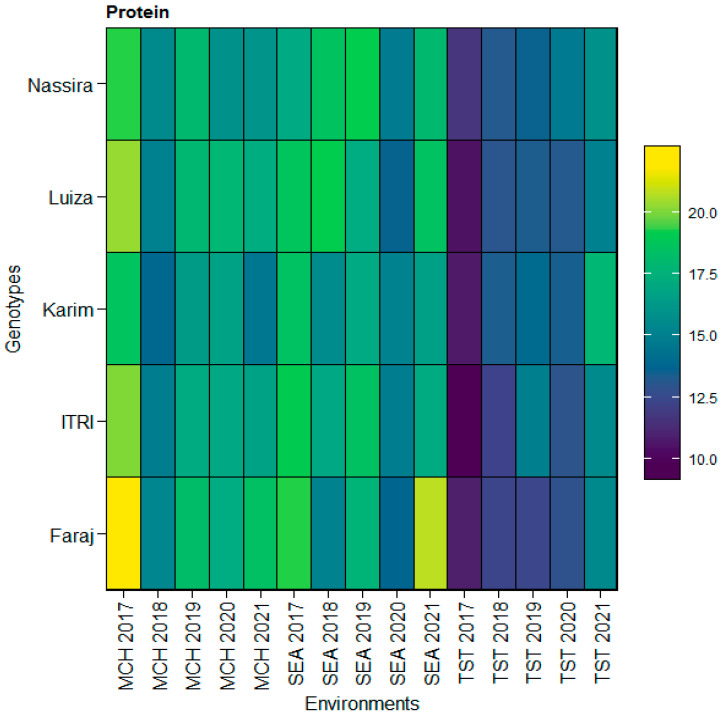
Heatmap illustrating variations in protein content across genotypes (Faraj, Itri, Karim, Luiza, and Nassira) and environments (SEA 2017–2021, MCH 2017–2021, and TST 2017–2021).

Upon integrating the insights from the heatmap analysis, we observed distinct patterns in protein content across the genotypes Faraj, Itri, Karim, Luiza, and Nassira in the environments SEA, MCH, and TST from 2017 to 2021. The heatmap illustrated the nuanced interactions between genotypes and environments, revealing areas of high protein content, especially in Faraj, across multiple environments. This corroborates the consistently high values observed in the SEA and MCH sites. Conversely, areas of lower protein content were also depicted, which aligned with the lower averages noted for Itri in the TST location. This visual representation enhanced our understanding of the intricate dynamics of protein content across different environments and genotypes, offering a holistic view of the variability and specific adaptations unique to each genotype, as depicted in Figure 7.

At the Tassaout (TST) location, quality traits, including protein content, gluten content, and baking strength, exhibited varied influences from genotypic and environmental factors, as comprehensively detailed in Table 9. The protein content displayed a non-significant effect from the genotype but was significantly influenced by environmental conditions (*p* < 0.001). The average protein content across the growing years fluctuated, starting from 10.70% during the 2016–2017 season, increasing to 12.88% in 2017–2018, and reaching a peak of 15.99% in 2020–2021. These variations in protein content over the years at TST, including the specific changes for each season, are meticulously documented in Table 9. This table clearly depicts how environmental factors across different growing seasons can significantly influence key quality traits like protein content in wheat.

The gluten content revealed a notable effect from genotype (*p* < 0.01) and was profoundly influenced by environmental conditions (*p* < 0.001). In contrast, the interaction between genotype and environment did not show significant effects (ns). The highest average gluten content was recorded in 2020–2021 at 32.14%, with Karim and Nassira showing elevated values of 34.85% and 33.29%, respectively. The lowest average gluten content was observed during the 2016–2017 growing season, at 23.84%, with Itri registering the lowest value of 21.29%.

Baking strength at TST displayed a highly significant genotypic effect (*p* < 0.001) and was also markedly influenced by the environment (*p* < 0.001). The genotype–environment interaction was non-significant (ns). During the 2016–2017 growing season, the average baking strength was 97.64 W, with Itri recording the lowest value of 53.47 W. A substantial increase was observed in the subsequent growing seasons, reaching its peak in 2017–2018 with an average of 226.65 W. Karim showed the highest value under this growing season, registering a baking strength of 265.46 W.

The data revealed a notable trend in protein content at the Tassaout (TST) location. TST, an irrigated site conventionally anticipated to be optimal for crop yield and quality conditions, has the lowest protein content among the three surveyed sites. In contrast, the SEA site, which had lower yields, showed a higher protein content. The data from SEA and TST illustrated an inverse relationship between yield and protein content. Such results suggest that while irrigation may enhance yield, particularly in traditionally arid regions like TST, it may simultaneously reduce grain protein concentration. This dynamic is crucial for breeding programs aiming to optimize wheat yield and protein content.

Expanding on the previous insights, we then focused on examining the intricate relationships between agronomic and quality traits within the framework of genotype–environment interactions.

### 3.8. Correlations among Agronomic and Quality Traits in the Context of Genotype–Environment Interactions

**Figure 8:**
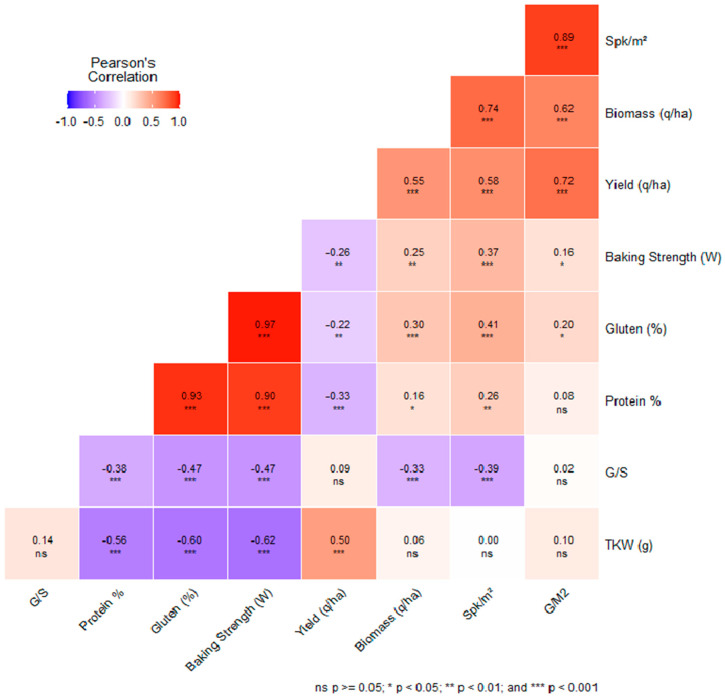
Pearson correlation coefficients and *p*-values among agronomic traits.

In the context of genotype–environment interactions, agronomic traits, such as yield, biomass, thousand kernel weight (TKW), spikes per square meter (Spk/m^2^), grain-to-straw ratio (G/S), and grain yield per square meter (G/m^2^), were evaluated alongside quality traits, such as protein percentage, gluten percentage, and baking strength. Pearson correlation coefficients and their respective *p*-values were calculated to gauge the relationships between these agronomic and quality traits (Figure 8).

For instance, a moderate positive correlation was observed between yield and biomass (r = 0.55, *p* < 0.05). This suggests that genotypes with increased biomass are generally associated with higher yields. Moreover, there was a negative correlation between yield and protein content (r = −0.33, *p* < 0.001), indicating that augmenting yield could potentially reduce protein concentration. The relationship between the density of spikes per square meter (Spk/m^2^) and biomass showed a significant positive trend (r = 0.74, *p* < 0.001), emphasizing that a rise in spike density is linked to greater biomass levels.

In addition, a prominent positive correlation was identified between grain yield per square meter (G/m^2^) and the aggregate yield (r = 0.72, *p* < 0.001). This relationship highlights that an increase in grain yield per square meter significantly boosts the total yield. As a result, G/m^2^ can be considered a reliable indicator for predicting yield outcomes.

In examining quality traits, a robust positive correlation was identified between protein and gluten contents (r = 0.93, *p* < 0.001), signifying a robust relationship between these two elements. This finding posits that a selection favoring higher protein content could concurrently augment gluten levels, a critical aspect for end-use quality.

Lastly, a robust positive correlation was noted between gluten content and baking strength (r = 0.97, *p* < 0.001). This observation underscores that gluten content significantly impacts baking strength, accentuating the significance of gluten quality in determining a genotype’s suitability for specific end applications.

The interactions and correlations presented here provide invaluable insights. The subsequent section aimed to contextualize these findings within the broader scientific discourse, offering a detailed understanding of the genotype–environment interaction effects on agronomic and quality traits.

## 4. Discussion

This comprehensive study, spanning five wheat-growing seasons across distinct agro-climatic zones in Morocco, has uncovered significant insights into genotype by environment (G × E) interactions, foundational to crop breeding programs [17,18,19]. This study’s meticulous design and robust statistical analysis have emphasized the differential performance of wheat genotypes across variable environmental conditions, echoing earlier findings [20,21,22,23]. The marked impact of environmental conditions on vital agronomic traits, such as yield, thousand kernel weight (TKW), and spikes per square meter (Spk/m^2^), highlights the profound influence of climatic variables on wheat performance, supporting previous findings [24,25,26].

The data revealed notable variations in yield performance that were location-specific. Particularly at Tassaout (TST), genotypes such as Luiza and Nassira displayed pronounced yield variations, underscoring the influence of genotype in this location. This finding aligns with the well-documented G × E interactions in wheat, emphasizing the role of specific environmental conditions in amplifying or mitigating genetic potential. While the genotypic effects were insignificant at SEA and MCH, the TST results clearly illustrated how different genotypes can exhibit varied performances under different environmental conditions. These insights correlate with previous research in this field, as reported by Eltaher et al. [27], Farokhzadeh et al. [28], and Sukumaran et al. [29], highlighting the critical impact of environmental factors on crop yield and performance. This variability underscores the need for tailored breeding and cultivation strategies considering genetic makeup and location-specific environmental conditions. The heatmap analysis provides a compelling visual representation of G × E interactions, mirroring the findings of Hacini et al. [30]. This approach is vital for grasping how different wheat genotypes respond to varied climatic conditions across Moroccan agro-climatic zones. Such insight is crucial for tailoring breeding programs to enhance wheat resilience and yield potential amidst changing environmental conditions, which is in agreement with the research of Ayed et al. [31] and Megahed et al. [32].

Expanding on genotype-specific responses, the pronounced genotypic effects on biomass, TKW, Spk/m^2^, and G/S at the TST location underscore the need for a genotype-specific approach in breeding programs. This is in line with the findings of Habash et al. [33], Tomić et al. [34], and Gagliardi et al. [35], which emphasize harnessing the inherent potential of different genotypes under variable environmental conditions. Looking ahead, delving into the physiological or genetic mechanisms underpinning the observed G × E interactions, akin to the findings of Niu et al. [36], Kebrom et al. [37], and Vicente et al. [38], would not only enhance the robustness of these findings but also provide actionable insights for breeding programs aiming at developing high-yielding, resilient wheat varieties suited to diverse Moroccan agro-climatic zones.

The methodologies employed, especially elucidating environmental discrimination and representativeness through biplot analysis, significantly advance our understanding of how different environments influence principal traits like yield and protein content in wheat. This approach resonates with earlier efforts focused on the genotype by environment interaction, pivotal in plant breeding programs, as highlighted by Mohammadi et al. [39], Sakin et al. [40], and Mohamed et al. [41]. The discrimination vs. representativeness biplot analysis highlighted the heterogeneity among the analyzed environments, supporting the idea that specific environments can significantly differentiate genotypes based on these traits. This in-depth analysis revealed the genotype Luiza’s adaptability across varied environments, echoing the notion supported by Verma et al. [42], Kamara et al. [43], and Ionut et al. [44] concerning the genetic potential inherent in several genotypes to withstand diverse environmental conditions.

Continuing with this inquiry, the environmental ranking through biplot analysis provides a hierarchical lens to evaluate the alignment of different environments with ideal conditions for yield and protein. This concept has been addressed in various studies, including those by Roostaei et al. [45], Tanin et al. [7], and Chairi et al. [46], which have explored the interactions between genotypes and environments, especially in durum wheat, and their implications for yield and other agronomic traits. Switching to a multi-metric assessment, the genotypic stability analysis exhibited commendable depth, employing notable stability metrics from the literature, as highlighted by Alemu Dabi et al. [47], Lin and Binns [48], and Shukla [14].

Adding a nuanced layer to our understanding of genotypic performance across environmental spectra, the mean vs. stability biplot analysis offers a visual interpretation of mean performance compared to stability. This concept has been addressed in various studies, including those by Martínez-Peña et al. [49] and Al-Sayaydeh et al. [50], which have explored the interactions between genotypes and environments, especially in durum wheat, and their implications for yield and other agronomic traits. The “Which Won Where/What” analysis provides a granular view of genotypic superiority under distinct environments, revealing the genotype-specific advantages that can be leveraged in breeding programs.

Building on the previously discussed analyses, the meticulous examination of the interaction between genotypes and environments on various quality traits like protein content, gluten content, and baking strength across different locations and growing seasons reaffirms the complexity of G × E interactions. This has been a well-established notion in the agronomic literature, as highlighted by Johnson et al. [51] and Plavšin et al. [52]. The observed trends underscore the compelling influence of environmental factors overriding genotypic factors in specific contexts. This corroborates previous findings where environmental factors played a pivotal role in the expression of quality traits in wheat, as highlighted by studies conducted by Vida et al. [53] and Pačuta et al. [54].

Delving deeper, the consistency of the Faraj genotype in maintaining a relatively high protein content across different environments, depicted in the heatmap, echoes the potential genetic resilience some genotypes exhibit towards environmental perturbations. While this is consistent with the theme that environmental factors and their interactions with genotypes play a significant role in the expression of quality traits in wheat, there is also evidence highlighted by studies conducted by Kyratzis et al. [55] and Bnejdi [56]. The significant decline in protein content over specific years reflects the environmental influence on this crucial quality trait. This observation resonates with previous findings highlighted by studies conducted by Zhang et al. [57], Nigro et al. [58], and Muqaddasi et al. [59].

Moreover, the significant genotypic effect observed for gluten content and baking strength across all locations emphasizes the genetic control over these traits. Studies such as Ruan et al. [60], Hao et al. [61], and Nazco et al. [62] have previously confirmed the genetic basis of gluten strength and its importance in durum wheat. Further, unveiling correlations among traits, the strong positive correlation between protein and gluten content, and gluten content and baking strength highlight the intertwined nature of these quality traits. This observation aligns with the findings of Kirouani et al. [63], Al-Khayri et al. [64], and Huertas-García et al. [65].

Similarly, the moderate to strong positive correlations between yield, biomass, and spikes per square meter underscore the interconnectedness of these agronomic traits, offering a fertile ground for exploring genotype selection and management practices to optimize yield and quality. This perspective is corroborated by the findings of Maich and Rienzo [66].

## 5. Conclusions

This extensive study, covering multiple growing seasons under diverse Moroccan agro-climatic zones, has yielded critical insights into genotype by environment (G × E) interactions, pivotal for refining wheat breeding strategies. Through meticulous experimental design and statistical analyses, we identified significant yet subtle variations in the performance of various wheat genotypes under different environmental conditions. Notably, significant genotypic effects on agronomic traits were evident under specific locations, while impacts on quality traits varied, highlighting the complexity and context-specific nature of genotypic influences. This underlines the need for nuanced breeding strategies, especially given the variation in genotypic responses influenced by environmental factors like erratic precipitation and drought, which are crucial in determining wheat yield in Morocco. To mitigate these environmental challenges, integrating technologies like desalination for consistent irrigation and tailored breeding strategies is vital for enhancing wheat production stability and ensuring agricultural resilience and food security. This study also emphasizes the importance of balancing yield improvement with quality maintenance, necessitating careful management within breeding programs. Insights from the biplot and heatmap analyses have deepened our understanding of the interactions between genotypes and environmental factors, supporting a breeding strategy that leverages genotypic strengths alongside environmental variations. The correlations among various agronomic and quality traits also lay the groundwork for strategic genotype selection and management to optimize yield and quality. This research significantly contributes to the wheat breeding discourse in Morocco. It aligns with global sustainable wheat breeding initiatives, emphasizing the critical importance of genotype–environment interplays under the changing climatic conditions.

## Figures and Tables

**Table 1:** Characteristics of experimental stations, including soil type, precipitation, geographical location, and altitude across five growing seasons.

Experimental Station	Soil Type	Precipitations (mm) for the Five Growing Seasons					Geographical Location		Altitude (Meters)
		2016–2017	2017–2018	2018–2019	2019–2020	2020–2021	Latitude	Longitude	
Sidi El Aidi (SEA)	Vertisol	290	505	210	242	467	33.12218° N	7.63315° W	235
Merchouch (MCH)	Cambisol	348	579	179	249	518	33.60499° N	6.71000° W	399
Tassaout (TST)	Alfisol	216	305	200	247	304	31.82021° N	7.43806° W	591

**Table 2:** Overview of cultivar registrations and their pedigree and breeding history.

Cultivar	Code	Year of Registration	Pedigree and Breeding History
Faraj	V1	2007	Hybrid Nassira, Qarmal, Lahn (ICARDA)
Itri	V2	2017	RISSA/GAN//POHO_1/3/PLATA_3//CREX/ALLA/x Karim
Karim	V3	1985	Bittern ‘S’ « JO’S’. AA”:S’//FG’S’ »
Luiza	V4	2011	RASCON_39/TILO_1
Nassira	V5	2003	INRA Selection on CIMMYT EII, 12 TA14/BD3//Isly # CF41530–1548

**Table 3:** Interaction analysis of genotype and environment on agronomic traits at Sidi El Aidi (SEA) across seasons.

Env (E): (Combinations of Location * Season)	Gen (G)	YLD (q/ha)	Biomass (q/ha)	TKW (g)	Spk/m^2^	G/S	G/m^2^
SEA 2017	V1	23.01 ^abc^	105 ^a^	37.3 ^bcdef^	324 ^ab^	25 ^a^	8300 ^ab^
	V2	23.21 ^abc^	77.5 ^a^	33.45 ^def^	284 ^abcd^	26 ^a^	7347 ^ab^
	V3	22.93 ^abc^	78.33 ^a^	43.1 ^abcde^	219 ^abcdef^	29 ^a^	6431 ^ab^
	V4	23.66 ^abc^	104.17 ^a^	33.9 ^def^	297 ^abc^	35 ^a^	9715 ^a^
	V5	24.65 ^abc^	111.67 ^a^	47.45 ^abc^	302 ^ab^	25 ^a^	7622 ^ab^
Mean		23.49	95.33	39.04	284	28	7883
SEA 2018	V1	48.82 ^a^	120.83 ^a^	49.13 ^ab^	284 ^abcd^	28 a	8092 ^ab^
	V2	44.78 ^ab^	106.67 ^a^	47.13 ^abc^	373 ^a^	21 ^a^	7690 ^ab^
	V3	38.28 ^abc^	103.33 ^a^	50.5 ^a^	213 ^abcdef^	29 ^a^	6143 ^ab^
	V4	40.95 ^abc^	122.5 ^a^	47 ^abc^	316 ^ab^	16 ^a^	5071 ^ab^
	V5	39.33 ^abc^	96.67 ^a^	45.88 ^abcd^	273 ^abcd^	14 ^a^	3677 ^ab^
Mean		42.43	110	47.93	289	22	6135
SEA 2019	V1	15 ^abc^	87.5 ^a^	35.4 ^cdef^	203 ^abcdefg^	28 ^a^	5549 ^ab^
	V2	16.73 ^abc^	94.17 ^a^	31.4 ^ef^	270 ^abcd^	27 ^a^	6596 ^ab^
	V3	22.75 ^abc^	97.5 ^a^	33 ^ef^	273 ^abcd^	26 ^a^	7155 ^ab^
	V4	27.78 ^abc^	105.83 ^a^	38.6 ^abcdef^	278 ^abcd^	26 ^a^	6869 ^ab^
	V5	3.32 ^c^	39.17 ^a^	34.49 ^def^	113 ^defg^	22 ^a^	2319 ^ab^
Mean		17.12	84.83	34.58	227	26	5698
SEA 2020	V1	8.58 ^abc^	95.37 ^a^	32.55 ^ef^	73 ^efg^	27 ^a^	1982 ^b^
	V2	5.49 ^bc^	82.3 ^a^	30.45 ^f^	49 ^efg^	25 ^a^	1204 ^b^
	V3	4.22 ^bc^	97.85 ^a^	31.35 ^ef^	32 ^g^	27 ^a^	842 ^b^
	V4	4.72 ^bc^	105.64 ^a^	30.25 ^f^	41 ^fg^	25 ^a^	1034 ^b^
	V5	8.51 ^abc^	82.7 ^a^	31.85 ^ef^	73 ^efg^	21 ^a^	1544 ^b^
Mean		6	93	31	54	25	1321
SEA 2021	V1	14.07 ^abc^	68.15 ^a^	31.2 ^ef^	111 ^defg^	32 ^a^	3289 ^ab^
	V2	17.76 ^abc^	50.85 ^a^	32.6 ^ef^	122 ^cdefg^	33 ^a^	3966 ^ab^
	V3	27.2 ^abc^	112.22 ^a^	40 ^abcdef^	224 ^abcde^	19 ^a^	5295 ^ab^
	V4	23.09 ^abc^	90.07 ^a^	35.6 ^cdef^	170 ^bcdefg^	28 ^a^	4717 ^ab^
	V5	23.26 ^abc^	83.3 ^a^	35.6 ^cdef^	184 ^bcdefg^	26 ^a^	4776 ^ab^
Mean		21	81	35	162	28	4409
Analysis of variance							
Gen (G)		ns	ns	*	ns	ns	ns
Env (E)		***	ns	***	***	ns	***
Gen (G) * Env (E)		ns	ns	*	**	ns	ns

Notes: Location: SEA = Sidi el Aidi. Agronomic terms: Env (E) = environment, Gen (G) = genotype, YLD = yield, TKW = thousand kernel weight, Spk/m^2^ = spikes per square meter, G/S = grains per spike, and G/m^2^ = grains per square meter. ANOVA: * = *p* ≤ 0.05, ** = *p* ≤ 0.01, *** = *p* ≤ 0.001, and ns = not significant. Means with identical letters are not significantly different at the 95% confidence interval (Tukey method).

**Table 4:** Interaction analysis of genotype and environment on agronomic traits at Merchouch (MCH) across seasons.

Env (E): (Combinations of Location * Season)	Gen (G)	YLD (q/ha)	Biomass (q/ha)	TKW (g)	Spk/m^2^	G/S	G/m^2^
MCH 2017	V1	34.3 ^bcdef^	247.5 ^ab^	34.9 ^abcd^	818 ^abcde^	12 ^d^	10,006 ^bcdefg^
	V2	35.46 ^bcdef^	229.5 ^ab^	33.35 ^abcd^	726 ^bcdefgh^	20 ^cd^	13,740 ^bcdef^
	V3	29.17 ^cdef^	216 ^ab^	39.05 ^abcd^	624 ^cdefghi^	16 ^d^	9785 ^bcdefg^
	V4	34.25 ^bcdef^	236.25 ^ab^	37.15 ^abcd^	726 ^bcdefgh^	19 ^cd^	13,794 ^bcdef^
	V5	34.64 ^bcdef^	258 ^a^	29.75 ^d^	875 ^abc^	19 ^cd^	16,262 ^abcd^
Mean		33.56	237.45	34.84	751	17	12,717
MCH 2018	V1	42.97 ^abcdef^	163.33 ^abcde^	46.75 ^ab^	240 ^hi^	34 ^a^	8046 ^cdefg^
	V2	56.6 ^abcd^	166.67 ^abcde^	45.75 ^abc^	281 ^fghi^	29 ^abc^	8168 ^cdefg^
	V3	44 ^abcdef^	148.33 ^abcde^	38.88 ^abcd^	278 ^fghi^	32 ^ab^	8710 ^cdefg^
	V4	44.47 ^abcdef^	137.5 ^abcde^	46.88 ^a^	281 ^fghi^	28 ^abc^	7698 ^cdefg^
	V5	25.73 ^cdef^	143.33 ^abcde^	44.38 ^abcd^	265 ^ghi^	19 ^cd^	5068 ^fg^
Mean		43	152	45	267	28	7538
MCH 2019	V1	35.81 ^bcdef^	154.17 ^abcde^	39 ^abcd^	338 ^defghi^	12 ^d^	4217 ^fg^
	V2	45.58 ^abcdef^	258.33 ^a^	39.15 ^abcd^	429 ^cdefghi^	13 ^d^	5449 ^efg^
	V3	40.47 ^abcdef^	208.33 ^abc^	37.8 ^abcd^	394 ^cdefghi^	13 ^d^	5108 ^efg^
	V4	53.1 ^abcde^	260 ^a^	37.45 ^abcd^	527 ^cdefghi^	14 ^d^	7117 ^defg^
	V5	46.85 ^abcdef^	210 ^abc^	40.95 ^abcd^	410 ^cdefghi^	20 ^cd^	8300 ^cdefg^
Mean		44	218	39	419	14	6038
MCH 2020	V1	23.1 ^def^	129.44 ^abcde^	39.7 ^abcd^	327 ^efghi^	17 ^cd^	5505 ^efg^
	V2	9.83 ^f^	38.31 ^e^	31.3 ^cd^	100 ^i^	22 ^abcd^	2395 ^g^
	V3	9.62 ^f^	47.1 ^de^	30.45 ^d^	116 ^i^	21 ^bcd^	2406 ^g^
	V4	14.22 ^f^	65.89 ^cde^	31.85 ^bcd^	178 ^i^	21 ^bcd^	4096 ^g^
	V5	16.22 ^ef^	108.38 ^bcde^	37.35 ^abcd^	308 ^efghi^	19 ^cd^	5729 ^efg^
Mean		14.6	77.83	34.13	205	20	4026
MCH 2021	V1	62.98 ^abc^	166.82 ^abcde^	39.15 ^abcd^	859 ^abcd^	17 ^cd^	14,693 ^abcde^
	V2	71.82 ^ab^	154.19 ^abcde^	35.1 ^abcd^	783 ^abcdefg^	22 ^abcd^	17,099 ^abc^
	V3	76.03 ^a^	190.06 ^abcd^	44.4 ^abcd^	940 ^ab^	21 ^bcd^	19,270 ^ab^
	V4	61.48 ^abc^	146.81 ^abcde^	38.55 ^abcd^	788 ^abcdef^	21 ^bcd^	16,554 ^abcd^
	V5	67.7 ^ab^	228.1 ^ab^	41.75 ^abcd^	1293 ^a^	19 ^cd^	23,895 ^a^
Mean		68	177.2	39.79	932	20	18,302
Analysis of Variance							
Gen (G)		ns	ns	ns	ns	ns	*
Env (E)		***	***	***	***	***	***
Gen (G) * Env (E)		ns	ns	ns	ns	**	ns

Notes: Location: MCH = Merchouch. Agronomic terms: Env (E) = environment, Gen (G) = genotype, YLD = yield, TKW = thousand kernel weight, Spk/m^2^ = spikes per square meter, G/S = grains per spike, and G/m^2^ = grains per square meter. ANOVA: * = *p* ≤ 0.05, ** = *p* ≤ 0.01, *** = *p* ≤ 0.001, and ns = not significant; Means with identical letters are not significantly different at the 95% confidence interval (Tukey method).

**Table 5:** Interaction analysis of genotype and environment on agronomic traits at Tassaout (TST) across seasons.

Env (E): (Combinations of Location * Season)	Gen (G)	YLD (q/ha)	Biomass (q/ha)	TKW (g)	Spk/m^2^	G/S	G/m^2^
TST 2017	V1	58.45 ^ab^	136.5 ^a^	58.35 ^a^	302 ^abc^	27 ^a^	8400 ^ab^
	V2	54.58 ^ab^	113.25 ^abcd^	46.1 ^abcde^	235 ^abc^	36 ^a^	8370 ^ab^
	V3	41.93 ^ab^	79.5 ^abcd^	53 ^ab^	192 ^abc^	31 ^a^	5770 ^ab^
	V4	58.22 ^ab^	85.5 ^abcd^	50 ^abcd^	197 ^abc^	34 ^a^	6488 ^ab^
	V5	51.97 ^ab^	111.75 ^bcd^	51.4 ^abc^	238 ^abc^	31 ^a^	7320 ^ab^
Mean		53.03	105.3	51.77	232	32	7270
TST 2018	V1	39.37 ^ab^	191.67 ^a^	47.75 ^abcde^	402 ^a^	17 ^a^	6661 ^ab^
	V2	50.73 ^ab^	83.33 ^abcd^	43.5 ^bcde^	362 ^ab^	26 ^a^	9447 ^ab^
	V3	56.62 ^ab^	152.5 ^abcd^	49.25 ^abcde^	383 ^a^	24 ^a^	9315 ^ab^
	V4	62 ^ab^	162.5 ^abcd^	47.38 ^abcde^	356 ^ab^	30 ^a^	10,562 ^ab^
	V5	66.57 ^a^	114.17 ^bcd^	49.25 ^abcde^	311 ^abc^	27 ^a^	8659 ^ab^
Mean		55.06	140.83	47.43	362	25	8929
TST 2019	V1	48.34 ^ab^	155 ^ab^	41.25 ^bcde^	373 ^ab^	23 ^a^	7803 ^ab^
	V2	30.79 ^ab^	107.5 ^abcd^	37.15 ^e^	167 ^bc^	33 ^a^	5513 ^ab^
	V3	40.54 ^ab^	134.17 ^abcd^	45.5 ^bcde^	265 ^abc^	26 ^a^	6774 ^ab^
	V4	38.66 ^ab^	116.67 ^bcd^	38.35 ^de^	208 ^abc^	28 ^a^	5873 ^ab^
	V5	39.62 ^ab^	86.67 ^cd^	44.6 ^bcde^	208 ^abc^	27 ^a^	5589 ^ab^
Mean		39.59	120	41.37	243	27	6310
TST 2020	V1	35.8 ^ab^	123.49 ^abc^	50.75 ^abcd^	257 ^abc^	23 ^a^	5889 ^ab^
	V2	25.65 ^ab^	59.75 ^abcd^	45.65 ^bcde^	135 ^c^	33 ^a^	4528 ^ab^
	V3	19.87 ^b^	60.73 ^abcd^	48.75 ^abcde^	130 ^c^	26 ^a^	3391 ^b^
	V4	30.5 ^ab^	76.42 ^bcd^	49.85 ^abcd^	165 ^bc^	28 ^a^	4620 ^ab^
	V5	23.08 ^ab^	54.54 ^cd^	51.8 ^abc^	122 ^c^	27 ^a^	3299 ^b^
Mean		26.98	74.99	49.36	159	27	4345
TST 2021	V1	34.62 ^ab^	130 ^abcd^	45.6 ^bcde^	254 ^abc^	26 ^a^	6661 ^ab^
	V2	62.77 ^ab^	194.31 ^abcd^	46.45 ^abcde^	289 ^abc^	41 ^a^	11,821 ^a^
	V3	24.15 ^ab^	144.92 ^abcd^	40.45 ^cde^	224 ^abc^	25 ^a^	5311 ^ab^
	V4	33.69 ^ab^	127.38 ^bcd^	47.9 ^abcde^	259 ^abc^	22 ^a^	5967 ^ab^
	V5	33.23 ^ab^	134.31 ^d^	47.4 ^abcde^	262 ^abc^	24 ^a^	6261 ^ab^
Mean		37.69	146.18	45.56	257	28	7204
Analysis of variance							
Gen (G)		ns	**	**	**	**	ns
Env (E)		***	***	***	***	ns	***
Gen (G) * Env (E)		ns	*	ns	ns	ns	ns

Notes: Location: TST = Tassaout. Agronomic terms: Env (E) = environment, Gen (G) = genotype, YLD = yield, TKW = thousand kernel weight, Spk/m^2^ = spikes per square meter, G/S = grains per spike, and G/m^2^ = grains per square meter. ANOVA: * = *p* ≤ 0.05, ** = *p* ≤ 0.01, *** = *p* ≤ 0.001, and ns = not significant. Means with identical letters are not significantly different at the 95% confidence interval (Tukey method).

**Table 6:** Comparative analysis of genotypic stability across multiple metrics.

Genotype	Francis Cumulative Values	GR (Francis)	Wricke’s Ecovalence (W)	GR (Wricke)	Shukla’s Stability Variance (σ^2^)	GR (Shukla)
Faraj (V1)	48	3	3,607,727	1	457	2
ITRI (V2)	48	3	5,763,770	5	580	5
Karim (V3)	34	1	4,630,654	3	400	1
Luiza (V4)	49	5	4,084,894	2	333	3
Nassira (V5)	46	2	5,041,188	4	602	4

**Table 7:** Interaction analysis of genotype and environment on protein content, gluten, and baking strength at Sidi El Aidi (SEA) across seasons.

Env (E): (Combinations of Location * Season)	Gen (G)	Protein (%)	Gluten (%)	Baking Strength (W)
SEA 2017	V1	19.46 ^ab^	49.63 ^a^	494.29 ^a^
	V2	19.17 ^ab^	49.9 ^a^	466.61 ^ab^
	V3	18.6 ^abcd^	48.39 ^ab^	454.14 ^ab^
	V4	18.8 ^abc^	48.3 ^abc^	451.57 ^abc^
	V5	17.12 ^bcdef^	42.03 ^abcdef^	378.54 ^bcdefgh^
Mean		18.63	47.65	449.03
SEA 2018	V1	15.18 ^def^	34.06 ^fg^	324.94 ^gh^
	V2	17.06 ^bcdef^	41.29 ^abcdefg^	370.62 ^bcdefgh^
	V3	15.62 ^cdef^	36.56 ^defg^	310.27 ^h^
	V4	19.24 ^ab^	48.44 ^ab^	453.43 ^ab^
	V5	18.69 ^abcd^	46.16 ^abcd^	426.9 ^abcdef^
Mean		17.16	41.3	377.23
SEA 2019	V1	17.73 ^abcde^	40.06 ^abcdefg^	392.15 ^bcdefgh^
	V2	18.57 ^abcd^	47.53 ^abc^	436.36 ^abcd^
	V3	17.12 ^bcdef^	42.9 ^abcdef^	385.59 ^bcdefgh^
	V4	17.23 ^bcdef^	44.11 ^abcdef^	412.67 ^abcdefg^
	V5	19.26 ^ab^	47.32 ^abc^	432.03 ^abcde^
Mean		17.98	44.38	411.76
SEA 2020	V1	13.8 ^f^	31.26 ^g^	306.14 ^h^
	V2	14.95 ^ef^	38.24 ^bcdefg^	351.07 ^cdefgh^
	V3	15.2 ^def^	38.13 ^cdefg^	343.56 ^defgh^
	V4	13.7 ^f^	35.07 ^efg^	328.13 ^fgh^
	V5	14.8 ^ef^	36.37 ^defg^	332.01 ^efgh^
Mean		14	36	332
SEA 2021	V1	20.9 ^a^	47.19 ^abc^	461.83 ^ab^
	V2	17.2 ^bcdef^	43.96 ^abcdef^	403.53 ^abcdefgh^
	V3	16.6 ^bcdef^	41.63 ^abcdef^	374.56 ^bcdefgh^
	V4	18.65 ^abcd^	47.7 ^abc^	446.14 ^abc^
	V5	18.1 ^abcde^	44.38 ^abcde^	405.47 ^abcdefgh^
Mean		18	45	418
Analysis of variance				
Gen (G)		ns	**	**
Env (E)		***	***	***
Gen (G) * Env (E)		***	***	***

Notes: Location: SEA = Sidi el Aidi. ANOVA: ** = *p* ≤ 0.01, *** = *p* ≤ 0.001, and ns = not significant. Means with identical letters are not significantly different at the 95% confidence interval (Tukey method).

**Table 8:** Interaction analysis of genotype and environment on protein content, gluten, and baking strength at Merchouch (MCH) across seasons.

Env (E): (Combinations of Location * Season)	Gen (G)	Protein (%)	Gluten (%)	Baking Strength (W)
MCH 2017	V1	22 ^a^	64.82 ^a^	622.86 ^a^
	V2	20.15 ^ab^	59.12 ^abc^	524.59 ^bc^
	V3	18.75 ^bcd^	55.42 ^cde^	494 ^cd^
	V4	20.4 ^ab^	63 ^ab^	582.47 ^ab^
	V5	19.5 ^abc^	56.45 ^bcd^	522.16 ^bc^
Mean		20.16	59.76	549.22
MCH 2018	V1	15.34 ^ghi^	36.54 ^jk^	330.03 ^hi^
	V2	14.88 ^hi^	36.03 ^jk^	297.86 ^i^
	V3	13.92 ^i^	32.43 ^k^	300.97 ^i^
	V4	15.1 ^hi^	36.71 ^jk^	307.25 ^i^
	V5	15.58 ^fghi^	36.98 ^ijk^	345.66 ^ghi^
Mean		15	36	316
MCH 2019	V1	18.27 ^bcdef^	49.6 ^defg^	466.3 ^cde^
	V2	17.21 ^cdefgh^	46.75 ^fg^	403.97 ^efg^
	V3	16.41 ^defghi^	44.13 ^fghi^	399.31 ^fg^
	V4	17.98 ^bcdefg^	50.51 ^def^	450.77 ^def^
	V5	18.1 ^bcdefg^	48.21 ^efg^	447.4 ^def^
Mean		18	48	434
MCH 2020	V1	17.25 ^cdefgh^	46.85 ^fg^	440.29 ^def^
	V2	17.05 ^cdefgh^	46.32 ^fgh^	400.17 ^fg^
	V3	16.75 ^cdefgh^	45.05 ^fgh^	407.7 ^efg^
	V4	17.9 ^bcdefg^	50.27 ^def^	448.53 ^def^
	V5	15.95 ^efghi^	42.48 ^ghij^	394.62 ^fg^
Mean		16.98	46.19	418.26
MCH 2021	V1	18.5 ^bcde^	50.2 ^def^	472.02 ^cd^
	V2	16.75 ^cdefgh^	45.5 ^fgh^	393.25 ^fgh^
	V3	14.6 ^hi^	39.27 ^hijk^	355.43 ^ghi^
	V4	17.3 ^cdefgh^	48.62 ^efg^	433.97 ^def^
	V5	16.05 ^defghi^	42.75 ^ghij^	397.29 ^fg^
Mean		16.64	45.27	410.39
Analysis of variance				
Gen (G)		***	***	***
Env (E)		***	***	***
Gen (G) * Env (E)		ns	*	***

Notes: Location: MCH = Merchouch. ANOVA: * = *p* ≤ 0.05, *** = *p* ≤ 0.001, and ns = not significant. Means with identical letters are not significantly different at the 95% confidence interval (Tukey method).

**Table 9:** Interaction analysis of genotype and environment on protein content, gluten, and baking strength at Tassaout (TST) across seasons.

Env (E): (Combinations of Location * Season)	Gen (G)	Protein (%)	Gluten (%)	Baking Strength (W)
TST 2017	V1	10.85 ^cde^	24.09 ^cd^	108.25 ^efgh^
	V2	9.8 ^e^	21.29 ^d^	53.47 ^h^
	V3	10.7 ^de^	23.86 ^cd^	89.79 ^fgh^
	V4	10.45 ^de^	23.52 ^cd^	80.97 ^gh^
	V5	11.7 ^bcde^	26.44 ^abcd^	155.73 ^bcdefg^
Mean		10.7	23.84	97.64
TST 2018	V1	12.37 ^bcde^	27.24 ^abcd^	215.95 ^abc^
	V2	12.22 ^bcde^	27.4 ^abcd^	185.05 ^abcde^
	V3	13.46 ^abcde^	30.73 ^abc^	265.46 ^a^
	V4	13.1 ^bcde^	30.42 ^abc^	222.97 ^abc^
	V5	13.26 ^abcde^	30.67 ^abc^	243.83 ^ab^
Mean		12.88	29.29	226.65
TST 2019	V1	12.41 ^bcde^	26.32 ^abcd^	166.04 ^bcdefg^
	V2	15.02 ^abcd^	31.58 ^abc^	153.49 ^bcdefg^
	V3	14.02 ^abcde^	29.81 ^abcd^	194.02 ^abcde^
	V4	13.38 ^abcde^	29.62 ^abcd^	166.57 ^bcdefg^
	V5	13.62 ^abcde^	30.14 ^abcd^	210.85 ^abcd^
Mean		13.69	29.49	178.19
TST 2020	V1	12.95 ^bcde^	25.8 ^bcd^	162.24 ^bcdefg^
	V2	13.05 ^bcde^	25.5 ^bcd^	123.38 ^defgh^
	V3	13.5 ^abcde^	26.27 ^abcd^	170.84 ^bcdefg^
	V4	13.25 ^abcde^	27.77 ^abcd^	155.41 ^bcdefg^
	V5	14.75 ^abcd^	30.96 ^abc^	216.52 ^abc^
Mean		13.5	27.26	165.68
TST 2021	V1	15.5 ^abc^	30.79 ^abc^	194.84 ^abcde^
	V2	15.55 ^ab^	30.16 ^abcd^	148.55 ^cdefg^
	V3	17.9 ^a^	34.85 ^a^	226.81 ^abc^
	V4	15.1 ^abcd^	31.6 ^abc^	178.55 ^abcdef^
	V5	15.9 ^ab^	33.29 ^ab^	232.98 ^abc^
Mean		15.99	32.14	196.34
Analysis of variance				
Gen (G)		ns	**	***
Env (E)		***	***	***
Gen (G) * Env (E)		ns	ns	ns

Notes: Location: TST = Tassaout. ANOVA: ** = *p* ≤ 0.01, *** = *p* ≤ 0.001, and ns = not significant. Means with identical letters are not significantly different at the 95% confidence interval (Tukey method).

## Data Availability

Data are available on request due to restrictions, e.g., privacy or ethical. The data presented in this study are available on request from the corresponding author. The data are not publicly available due to ongoing analyses and intended use in future publications.

## References

[1] Rossini F., Provenzano M.E., Sestili F., Ruggeri R. (2018). Synergistic effect of sulfur and nitrogen in the organic and mineral fertilization of durum wheat: Grain yield and quality traits in the Mediterranean environment. Agronomy.

[2] Beres B.L., Rahmani E., Clarke J.M., Grassini P., Pozniak C.J., Geddes C.M., Porker K.D., May W.E., Ransom J.K. (2020). A systematic review of durum wheat: Enhancing production systems by exploring genotype, environment, and management (G × E × M) synergies. Front. Plant Sci..

[3] Jaisi S., Thapa A., Poudel M.R. (2021). Study of Correlation Coefficient and Path Analysis Among Yield Parameters of Wheat: A Review. INWASCON Technol. Mag. (I-TECH MAG).

[4] Turuspekov Y., Baibulatova A., Yermekbayev K., Tokhetova L., Chudinov V., Sereda G., Ganal M., Griffiths S., Abugalieva S. (2017). GWAS for plant growth stages and yield components in spring wheat (*Triticum aestivum* L.) harvested in three regions of Kazakhstan. BMC Plant Biol..

[5] Mohammadi R., Armion M., Zadhasan E., Ahmadi M.M., Amri A. (2018). The use of AMMI model for interpreting genotype × environment interaction in durum wheat. Exp. Agric..

[6] Mohammadi R., Armion M., Sadeghzadeh D., Amri A., Nachit M. (2011). Analysis of genotype-by-environment interaction for agronomic traits of durum wheat in Iran. Plant Prod. Sci..

[7] Tanin M.J., Sharma A., Saini D.K., Singh S., Kashyap L., Srivastava P., Mavi G., Kaur S., Kumar V., Kumar V. (2022). Ascertaining yield and grain protein content stability in wheat genotypes having the Gpc-B1 gene using univariate, multivariate, and correlation analysis. Front. Genet..

[8] Kendal E. (2019). Comparing durum wheat cultivars by genotype × yield × trait and genotype × trait biplot method. Chil. J. Agric. Res..

[9] Slim A., Piarulli L., Chennaoui Kourda H., Rouaissi M., Robbana C., Chaabane R., Pignone D., Montemurro C., Mangini G. (2019). Genetic structure analysis of a collection of Tunisian durum wheat germplasm. Int. J. Mol. Sci..

[10] Newton I. (2014). Minitab Cookbook.

[11] Ryan B.F., Joiner B.L., Cryer J.D. (2012). MINITAB Handbook: Update for Release.

[12] Francis T., Kannenberg L. (1978). Yield stability studies in short-season maize. I. A descriptive method for grouping genotypes. Can. J. Plant Sci..

[13] Wricke G. (1962). Uber eine methode zur erfassung der okologischen streubreite in feldversucen. Z. Pflanzenzüchtung.

[14] Shukla G. (1972). Some statistical aspects of partitioning genotype-environmental components of variability. Heredity.

[15] Wang Y., Mi J. (2019). Applying statistical methods to library data analysis. Ser. Libr..

[16] Tonk F.A., Ilker E., Tosun M. (2011). Evaluation of genotype x environment interactions in maize hybrids using GGE biplot analysis. Crop Breed. Appl. Biotechnol..

[17] Lopez-Cruz M., Crossa J., Bonnett D., Dreisigacker S., Poland J., Jannink J.-L., Singh R.P., Autrique E., de los Campos G. (2015). Increased prediction accuracy in wheat breeding trials using a marker × environment interaction genomic selection model. G3 Genes Genomes Genet..

[18] El Haddad N., Sanchez-Garcia M., Visioni A., Jilal A., El Amil R., Sall A.T., Lagesse W., Kumar S., Bassi F.M. (2021). Crop wild relatives crosses: Multi-location assessment in durum wheat, barley, and lentil. Agronomy.

[19] Bassi F., Sanchez-Garcia M. (2017). Adaptation and stability analysis of ICARDA durum wheat elites across 18 countries. Crop Sci..

[20] Mulugeta B., Tesfaye K., Geleta M., Johansson E., Hailesilassie T., Hammenhag C., Hailu F., Ortiz R. (2022). Multivariate analyses of Ethiopian durum wheat revealed stable and high yielding genotypes. PLoS ONE.

[21] Anastasi U., Corinzia S.A., Cosentino S.L., Scordia D. (2019). Performances of durum wheat varieties under conventional and no-chemical input management systems in a semiarid Mediterranean environment. Agronomy.

[22] Jokar F., Karimizadeh R., Masoumiasl A., Fahliani R.A. (2018). Canopy temperature and chlorophyll content are effective measures of drought stress tolerance in durum wheat. Not. Sci. Biol..

[23] van der Bom F.J., Williams A., Raymond N.S., Alahmad S., Hickey L.T., Singh V., Bell M.J. (2023). Root angle, phosphorus, and water: Interactions and effects on durum wheat genotype performance in drought-prone environments. Plant Soil.

[24] Arjona J.M., Royo C., Dreisigacker S., Ammar K., Villegas D. (2018). Effect of Ppd-A1 and Ppd-B1 allelic variants on grain number and thousand kernel weight of durum wheat and their impact on final grain yield. Front. Plant Sci..

[25] Negisho K., Shibru S., Matros A., Pillen K., Ordon F., Wehner G. (2022). Association mapping of drought tolerance indices in Ethiopian durum wheat (*Triticum turgidum* ssp. durum). Front. Plant Sci..

[26] Rabti A.-b., Mekaoussi R., Fellahi Z.E.A., Hannachi A., Benbelkacem A., Benmahammed A., Bouzerzour H. (2020). Characterization of old and recent durum wheat [*Triticum turgidum* (L.) Tell. convar. durum (Desf.) Mackey] varieties assessed under South Mediterranean conditions. Egypt. J. Agron..

[27] Eltaher S., Baenziger P.S., Belamkar V., Emara H.A., Nower A.A., Salem K.F., Alqudah A.M., Sallam A. (2021). GWAS revealed effect of genotype × environment interactions for grain yield of Nebraska winter wheat. BMC Genom..

[28] Farokhzadeh S., Shahsavand Hassani H., Mohammadi-Nejad G., Zinati Z. (2022). Evaluation of grain yield stability of tritipyrum as a novel cereal in comparison with triticale lines and bread wheat varieties through univariate and multivariate parametric methods. PLoS ONE.

[29] Sukumaran S., Jarquin D., Crossa J., Reynolds M. (2018). Genomic-enabled prediction accuracies increased by modeling genotype × environment interaction in durum wheat. Plant Genome.

[30] Hacini N., Djelloul R., Desclaux D. (2022). Study of genotype × environment interactions and agro-technological behavior of durum wheat varieties applied in different agro-climatic zones of algeria. Plant Arch..

[31] Ayed S., Bouhaouel I., Othmani A., Bassi F.M. (2021). Use of wild relatives in durum wheat (*Triticum turgidum* L. var. durum Desf.) breeding program: Adaptation and stability in context of contrasting environments in Tunisia. Agronomy.

[32] Megahed E.M., Awaad H.A., Ramadan I.E., Abdul-Hamid M.I., Sweelam A.A., El-Naggar D.R., Mansour E. (2022). Assessing performance and stability of yellow rust resistance, heat tolerance, and agronomic performance in diverse bread wheat genotypes for enhancing resilience to climate change under Egyptian conditions. Front. Plant Sci..

[33] Habash D.Z., Baudo M., Hindle M., Powers S.J., Defoin-Platel M., Mitchell R., Saqi M., Rawlings C., Latiri K., Araus J.L. (2014). Systems responses to progressive water stress in durum wheat. PLoS ONE.

[34] Tomić J.M., Torbica A.M., Popović L.M., Rakita S.M., Živančev D.R. (2015). Breadmaking potential and proteolytic activity of wheat varieties from two production years with different climate conditions. Food Feed Res..

[35] Gagliardi A., Carucci F., Masci S., Flagella Z., Gatta G., Giuliani M.M. (2020). Effects of genotype, growing season and nitrogen level on gluten protein assembly of durum wheat grown under mediterranean conditions. Agronomy.

[36] Niu N., Arief V.N., DeLacy I.H., Lush D., Sheppard J., Zhang G., Dieters M.J. (2010). Genetic gain in yield and protein over two cycles of a wheat recurrent selection program. Breed. Sci..

[37] Kebrom T.H., Chandler P.M., Swain S.M., King R.W., Richards R.A., Spielmeyer W. (2012). Inhibition of tiller bud outgrowth in the tin mutant of wheat is associated with precocious internode development. Plant Physiol..

[38] Vicente R., Pérez P., Martínez-Carrasco R., Feil R., Lunn J.E., Watanabe M., Arrivault S., Stitt M., Hoefgen R., Morcuende R. (2016). Metabolic and transcriptional analysis of durum wheat responses to elevated CO_2_ at low and high nitrate supply. Plant Cell Physiol..

[39] Mohammadi R., Sadeghzadeh B., Ahmadi M.M. (2020). Evaluation of genotype × environment interaction in durum wheat (*Triticum turgidum* var. durum L.) regional yield trials. Iran. J. Crop Sci..

[40] Sakin M.A., Akinci C., Duzdemir O., Donmez E. (2011). Assessment of genotype x environment interaction on yield and yield components of durum wheat genotypes by multivariate analyses. Afr. J. Biotechnol..

[41] Mohamed M.M., Darwish M., El-Rady A., Ghalab E., Elfanah A. (2022). Estimation of AMMI and GGE biplots for some bread and durum wheat genotypes. J. Plant Prod..

[42] Verma A., Chatrath R., Singh G. (2020). Adaptability of wheat genotypes under multi-environment trials for Northern Hills Zone. Int. J. Bio-Resour. Stress Manag..

[43] Kamara M.M., Rehan M., Mohamed A.M., El Mantawy R.F., Kheir A.M., Abd El-Moneim D., Safhi F.A., ALshamrani S.M., Hafez E.M., Behiry S.I. (2022). Genetic potential and inheritance patterns of physiological, agronomic and quality traits in bread wheat under normal and water deficit conditions. Plants.

[44] Ionut R., Hiriscau D., Kadar R., Berindean I. (2022). Phenotypic response of spring wheat perspective line genotypes to different environmental conditions. Life Sci. Sustain. Dev..

[45] Roostaei M., Jafarzadeh J., Roohi E., Nazary H., Rajabi R., Mohammadi R., Khalilzadeh G.R., Seif F., Mirfatah S.M.M., Amiri S.S. (2022). Genotype × environment interaction and stability analyses of grain yield in rainfed winter bread wheat. Exp. Agric..

[46] Chairi F., Aparicio N., Serret M.D., Araus J.L. (2020). Breeding effects on the genotype × environment interaction for yield of durum wheat grown after the Green Revolution: The case of Spain. Crop J..

[47] Dabi A., Alemu G., Sime B., Geleta N., Delessa A., Solomon T., Zegaye H., Asnake D., Asefa B., Duga R. (2023). Genotype × Environment Interaction and Stability Analysis Using GGE Biplot for Grain Yield of Bread Wheat (*Triticum aestivum*) Genotypes under Low Moisture Stress Areas of Ethiopia. Int. J. Bio-Resour. Stress Manag..

[48] Lin C.-S., Binns M.R. (1988). A superiority measure of cultivar performance for cultivar × location data. Can. J. Plant Sci..

[49] Martínez-Peña R., Schlereth A., Höhne M., Encke B., Morcuende R., Nieto-Taladriz M.T., Araus J.L., Aparicio N., Vicente R. (2022). Source-sink dynamics in field-grown durum wheat under contrasting nitrogen supplies: Key role of non-foliar organs during grain filling. Front. Plant Sci..

[50] Al-Sayaydeh R., Shtaya M., Qubbaj T., Al-Rifaee M., Alabdallah M., Migdadi O., Gammoh I., Al-Abdallat A. (2023). Performance and Stability Analysis of Selected Durum Wheat Genotypes Differing in Their Kernel Characteristics. Plants.

[51] Johnson M., Kumar A., Oladzad-Abbasabadi A., Salsman E., Aoun M., Manthey F.A., Elias E.M. (2019). Association mapping for 24 traits related to protein content, gluten strength, color, cooking, and milling quality using balanced and unbalanced data in durum wheat [*Triticum turgidum* L. var. durum (Desf).]. Front. Genet..

[52] Plavšin I., Gunjača J., Šimek R., Novoselović D. (2021). Capturing GEI patterns for quality traits in biparental wheat populations. Agronomy.

[53] Vida G., Cséplő M., Rakszegi M., Bányai J. (2021). Effect of multi-year environmental and meteorological factors on the quality traits of winter durum wheat. Plants.

[54] Pacuta V., Rasovsky M., Michalska-Klimczak B., Wyszynski Z. (2021). Grain yield and quality traits of durum wheat (*Triticum durum* Desf.) treated with seaweed-and humic acid-based biostimulants. Agronomy.

[55] Kyratzis A.C., Pallides A., Katsiotis A. (2022). Investigating stability parameters for agronomic and quality traits of durum wheat grown under Mediterranean conditions. Agronomy.

[56] Bnejdi F., Gazzah M.E. (2010). Epistasis and genotype-by-environment interaction of grain protein content in durum wheat. Genet. Mol. Biol..

[57] Zhang X., Ma X., Li Y., Ju H. (2022). Geographical detector-based wheat quality attribution under genotype, environment, and crop management frameworks. Front. Environ. Sci..

[58] Nigro D., Fortunato S., Giove S.L., Paradiso A., Gu Y.Q., Blanco A., De Pinto M.C., Gadaleta A. (2016). Glutamine synthetase in durum wheat: Genotypic variation and relationship with grain protein content. Front. Plant Sci..

[59] Muqaddasi Q.H., Brassac J., Ebmeyer E., Kollers S., Korzun V., Argillier O., Stiewe G., Plieske J., Ganal M.W., Röder M.S. (2020). Prospects of GWAS and predictive breeding for European winter wheat’s grain protein content, grain starch content, and grain hardness. Sci. Rep..

[60] Ruan Y., Yu B., Knox R.E., Singh A.K., DePauw R., Cuthbert R., Zhang W., Piche I., Gao P., Sharpe A. (2020). High density mapping of quantitative trait loci conferring gluten strength in Canadian durum wheat. Front. Plant Sci..

[61] Hao S., Lou H., Wang H., Shi J., Liu D., Tao J., Miao S., Pei Q., Yu L., Wu M. (2022). Genome-wide association study reveals the genetic basis of five quality traits in Chinese wheat. Front. Plant Sci..

[62] Nazco R., Peña R.J., Ammar K., Villegas D., Crossa J., Royo C. (2014). Durum wheat (*Triticum durum* Desf.) Mediterranean landraces as sources of variability for allelic combinations at Glu-1/Glu-3 loci affecting gluten strength and pasta cooking quality. Genet. Resour. Crop Evol..

[63] Kirouani A., Taghouti M., Boukhalfoun L., Henkrar F., Udupa S. (2020). Bread making possibility of Algerian durum wheat (*Triticum turgidum* L. var. Durum) varieties. J. Fundam. Appl. Sci..

[64] Al-Khayri J.M., Alshegaihi R.M., Mahgoub E.I., Mansour E., Atallah O.O., Sattar M.N., Al-Mssallem M.Q., Alessa F.M., Aldaej M.I., Hassanin A.A. (2023). Association of High and Low Molecular Weight Glutenin Subunits with Gluten Strength in Tetraploid Durum Wheat (*Triticum turgidum* spp. Durum L.). Plants.

[65] Huertas-García A.B., Guzmán C., Ibba M.I., Rakszegi M., Sillero J.C., Alvarez J.B. (2023). Processing and bread-making quality profile of spanish spelt wheat. Foods.

[66] Maich R., Di Rienzo J. (2014). Genotype × tillage interaction in a recurrent selection program in wheat. Cereal Res. Commun..

